# Risk factor analysis and establishment of a predictive model for epilepsy comorbid with depression

**DOI:** 10.1371/journal.pone.0331441

**Published:** 2025-09-02

**Authors:** Yiming Sun, Zhangyi Wu, Liang Guo, Wei Wang, Xiaowen Ma

**Affiliations:** 1 Department of Neurosurgery, Tongde Hospital of Zhejiang Province, Hangzhou, Zhejiang, China; 2 Department of Breast Surgery, Hangzhou Women’s Hospital, Hangzhou, Zhejiang, China; Niigata University of Health and Welfare: Niigata Iryo Fukushi Daigaku, JAPAN

## Abstract

**Objective:**

This study aims to utilize our hospital’s existing Stereo Electroencephalography (SEEG) examination results combined with other clinical data to systematically analyze the risk factors for epilepsy comorbid with depression, and to establish a model for predicting the risk of developing depression in epilepsy patients. Clinically, this model can be used to predict the risk of comorbid depression in epilepsy patients, thereby enhancing the identification of this condition and providing a theoretical basis for proactive intervention in depressive symptoms among epilepsy patients.

**Methods:**

A retrospective analysis was conducted on the clinical data of patients diagnosed with epilepsy in the Department of Neurosurgery at Tongde Hospital Of Zhejiang Province from 01/01/2020–31/12/2024, all of whom underwent Electroencephalography (EEG) examinations. According to the C-NDDI-E scores and clinical manifestations, the epilepsy patients were divided into an epilepsy with comorbid depression group (study group) and epilepsy without depression group (control group). Univariate analysis was performed using SPSS 26.0 software to screen for potential factors contributing to depression comorbid with epilepsy. Variables with a univariate P ≤ 0.05 were entered into a linear Lasso regression analysis. Those with statistical significance were then used to construct a nomogram model for predicting the risk of depression comorbid with epilepsy using R software.

**Results:**

A total of 152 epilepsy patients were enrolled, including 43 in the study group and 109 in the control group. Univariate analysis showed statistically significant (P < 0.05) differences between the groups in terms of age, employment status, marital status, age of onset, frequency of epileptic seizures, type of drug treatment, scalp EEG-determined epileptogenic zone, SEEG-determined epileptogenic zone, and Activities of Daily Living (ADL) score. Lasso regression analysis revealed that marital status (p = 0.0008), Enrollment age (OR = 0.9152, P = 0.0003, 95% CI: 0.8673–0.9562), frequency of epileptic seizures (OR =5.9946, P = 0.0030, 95% CI: 1.8952–20.6541), type of drug treatment (OR = 44.4062, P = 0.0157, 95% CI: 1.3629–15.6702), SEEG results indicating the epileptogenic zone (hippocampal onset: OR = 12.3489, P = 0.0026, 95% CI: 2.5902–70.9811), and ADL score (OR = 0.9358, P = 0.0314, 95% CI: 0.8785–0.9930) were independent risk factors for depression comorbid with epilepsy. The area under the ROC curve (AUC) was 0.895, indicating strong discriminative ability and high predictive accuracy.

**Conclusion:**

Independent risk factors for depression comorbid with epilepsy include: hippocampal origin of epilepsy as identified by SEEG, unstable marital status, younger age at the time of enrollment, higher frequency of epileptic seizures (>4 times/month), use of specific anti-seizure medications (such as topiramate, phenobarbital, levetiracetam, and perampanel), and lower activities of daily living (ADL) scores. The nomogram model established based on these factors performs well in relatively accurately predicting the risk of depression comorbid with epilepsy. This facilitates early identification of high-risk patients in clinical practice, enabling timely interventions to prevent the severe consequences of depressive episodes, improving patient adherence to epilepsy treatment, and emphasizing the link between psychological and neuroscientific aspects in epilepsy management to foster interdisciplinary collaboration for more comprehensive patient care.

## Introduction

Common comorbidities of epilepsy include neurological, psychiatric, and somatic diseases with psychiatric comorbidities having a high lifetime prevalence exceeding 30% [1,2]. Psychiatric comorbidities in epilepsy encompass depression, anxiety, attention deficit hyperactivity disorder, autism, intellectual disability, anorexia nervosa, bipolar disorder, etc. [3]. Among these, depression is the most common [4–6]. Approximately 20%–55% of epilepsy patients experience comorbid depression [7–9]. In one study, depression prevalence in epilepsy patients was 11% (55/499), exceeding general population rates [10], substantially elevating suicide risk in this population. A study conducted in Denmark found that 492 (2.32%) individuals who committed suicide had epilepsy compared with 3140 (0.74%) individuals without epilepsy, corresponding to a three times higher risk (RR 3.17, 95% CI 2.88–3.50; p < 0.0001). In individuals with epilepsy, the highest risk of suicide was especially high in those with a history of comorbid psychiatric disease (Relative Risk (RR) 29.2, 95% CI 16.4–51.9; p < 0.0001) [6].

Risk factors for depression comorbid with epilepsy include psychosocial factors, neurobiological factors, treatment factors, and disease-related factors. Psychosocial factors involve gender, low education level, economic pressure, social isolation and stigma [11–13]. Neurobiological factors emphasize abnormalities in brain structure and function—especially in regions such as the hippocampus, amygdala, orbitofrontal cortex, and anterior cingulate cortex [11]. The most commonly associated factor is the frequency of epileptic seizures; most studies have shown a significant positive correlation between seizure frequency and depression, with each additional seizure per month increasing the risk of depression by 38% [13]. Among all epilepsy types, temporal lobe epilepsy has the highest prevalence of depression [11]. However, the risk factors for epilepsy comorbid with depression are not yet fully understood, and the current status of diagnosis and treatment for this condition in China remains suboptimal. A cross-sectional study reported a 24.1% prevalence of depression among 407 epilepsy patients, yet only 6.2% had been previously diagnosed, highlighting a major gap in clinical recognition and screening [14]. Although several predictive models have been developed using clinical or psychosocial variables, many suffer from methodological limitations such as small sample sizes, absence of external validation, and lack of incorporation of neurophysiological data. For example, Aljafen et al. (2024) constructed a logistic regression-based model targeting depression in patients with temporal lobe epilepsy with hippocampal sclerosis [15]; however, it did not include functional or electrophysiological parameters such as SEEG findings, limiting its generalizability and predictive accuracy [16]. To address these gaps, we developed a predictive model for depression in epilepsy based on our hospital’s SEEG data integrated with demographic and clinical variables, aiming to enhance risk stratification and facilitate timely intervention for high-risk patients.

## Materials and methods

### Study subjects

From 01/01/2020–31/12/2024, patients diagnosed with epilepsy according to the International League Against Epilepsy criteria were recruited from the Department of Neurosurgery at our hospital. Among these, 152 epilepsy patients who underwent evaluation with the Chinese version of the C-NDDI-E (the Chinese version of the Center for Epidemiologic Studies Depression Scale, CES-D) [17]and EEG or SEEG examination were included in the study, comprising 87 males and 65 females. General demographic data and disease-related information were recorded.

The NDDI-E is a 6-item self-report screening tool designed to identify major depressive episodes in epilepsy patients. It assesses depressive symptoms over the past two weeks using a Likert scale (1–4). Developed to minimize confounding effects of antiepileptic drug side effects and epilepsy-related cognitive issues. In the Arabic version, a cutoff score > 15 demonstrated high sensitivity (93.33%) and specificity (94.44%) for major depression diagnosis [18]. This instrument was validated for Chinese people with epilepsy with a suggested cutoff point of>12, with a sensitivity of 0.926 and a specificity of 0.804. In this study population the CNDDI-E adopted>12 as the cutoff score [19]. Subjects with a score greater than 12 were considered to be depressed [19].

Inclusion Criteria: 1. Aged 15–59 years. 2. Underwent EEG/video-EEG/SEEG in the Neurosurgery Department. 3. Epilepsy diagnosis per ILAE criteria, with ≥2-year duration. 4. Ability to complete the C-NDDI-E assessment.

Exclusion Criteria: 1. Impaired consciousness or comprehension precluding C-NDDI-E completion. 2. Systemic malignancies. 3. Incomplete clinical data. 4. Depression secondary to brain tumors, trauma, etc. 5. Comorbid functional/organic mental disorders.6. Pregnancy/lactation. 7. History of substance/alcohol abuse.

Group Allocation: Epilepsy with depression (study group): C-NDDI-E score >12. Epilepsy without depression (control group): C-NDDI-E score ≤12.

This study was approved by the Ethics Committee of Tongde Hospital of Zhejiang Province (Approval Code: 2025 Yan No. 094). See Supporting Information S1–S2 File for details. This study complied with the requirements for Human Participants Research; details are provided in the Supporting Information S3 File.

### Methods

We began collecting patient data for this study on 01/03/2025. This was a retrospective study. Patients diagnosed with epilepsy between 01/01/2020 and 31/12/2024, were consecutively selected from hospital records. No specific proportion of patients with comorbid depression was targeted a priori. Relevant data were collected for each subject, including: Age at enrollment, Gender, Body Mass Index (BMI), Education level (1: below primary school, 2: primary school, 3: junior high school, 4: high school, 5: college, 6: junior college, 7: bachelor’s, 8: master’s and above), Employment status (1: unemployed, 2: freelance/flexible employment, 3: civil servant or other stable employment, 4: self-employed, 5: student, 6: retired), Marital status (unmarried, married, divorced or widowed), Age of onset of epilepsy, Frequency of epileptic seizures per month (≤4 times; > 4 times), Drug treatment (1. Treatment including topiramate, phenobarbital, levetiracetam, perampanel; 2. Without the aforementioned drugs), scalp EEG-determined epileptogenic zone (1. Temporal lobe, 2. Frontal lobe, 3. Parietal lobe, 4. Occipital lobe), Number of epileptogenic zone (1: single focus, 2: multiple foci), SEEG-determined epileptogenic zone (hippocampal; non-hippocampal), ADL score, Family history of epilepsy or depression.

To increase the sample size, we included patients aged 15–59 years. Although participants aged 15–17 are considered adolescents, validated depression screening tools such as the NDDI-E is applicable and widely used in this age group, supporting the inclusion of older adolescents in the study. Rationale for Setting 59 Years as the Upper Age Limit: Unique Characteristics of Elderly Epilepsy Patients: In patients aged ≥60 years, epilepsy is predominantly secondary to cerebrovascular disorders, brain tumors, or neurodegenerative diseases (e.g., Alzheimer’s disease) [20]. Excluding patients ≥60 years minimizes age-related confounding comorbidities, ensuring cohort homogeneity and enhancing the generalizability of findings to young/middle-aged epilepsy populations.

This study focused on four Anti-Seizure Medication—topiramate [16,20,21], levetiracetam [22], perampane [23], and phenobarbita [24]—because prior clinical evidence suggests these particular drugs may increase susceptibility to depression or worsen depressive symptoms in epilepsy patients.

In our study, we focused on distinguishing hippocampal-onset epilepsy from other types based on SEEG data, as hippocampal involvement is strongly associated with psychiatric comorbidities, particularly depression, as the hippocampus regulates emotion via limbic circuits, hippocampal disfunction may disrupt these pathways, potentially explaining epilepsy-depression comorbidity [11,14].

**Fig 1:**
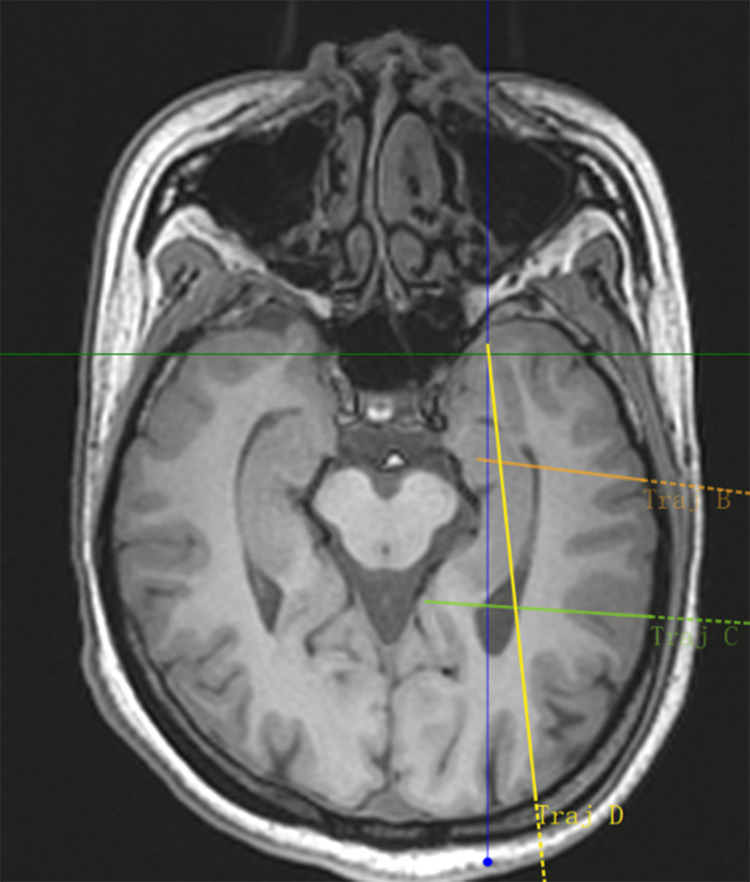
MRI T1 sequence transverse view.

**Fig 2:**
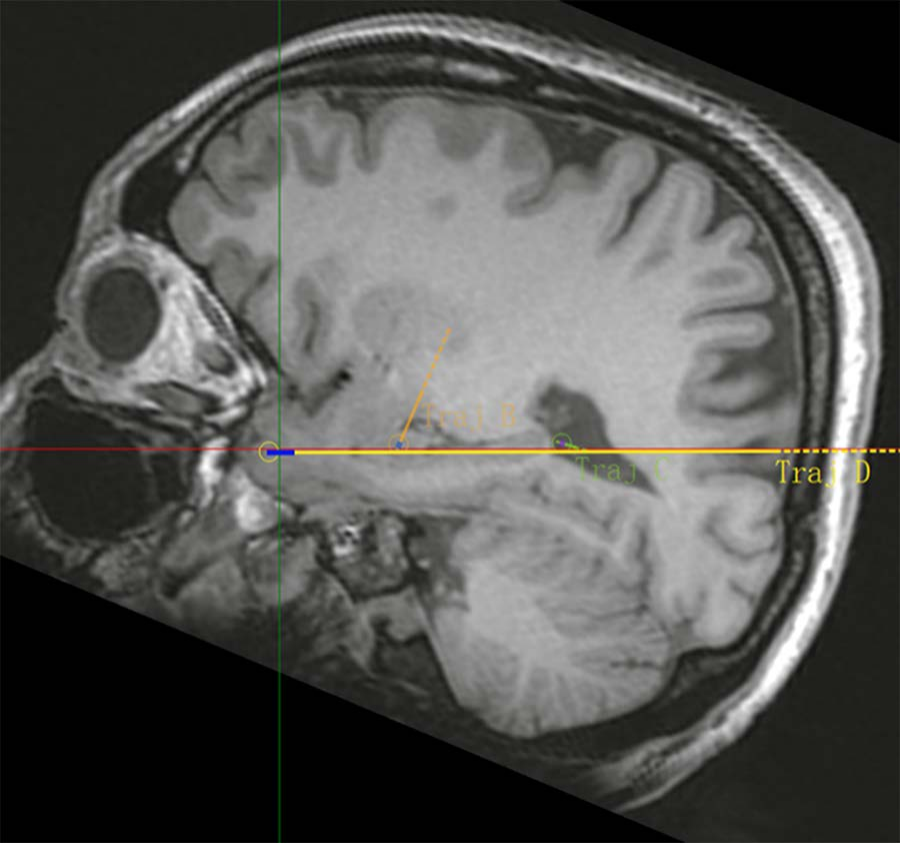
MRI T1 sequence sagittal view.

**Fig 3:**
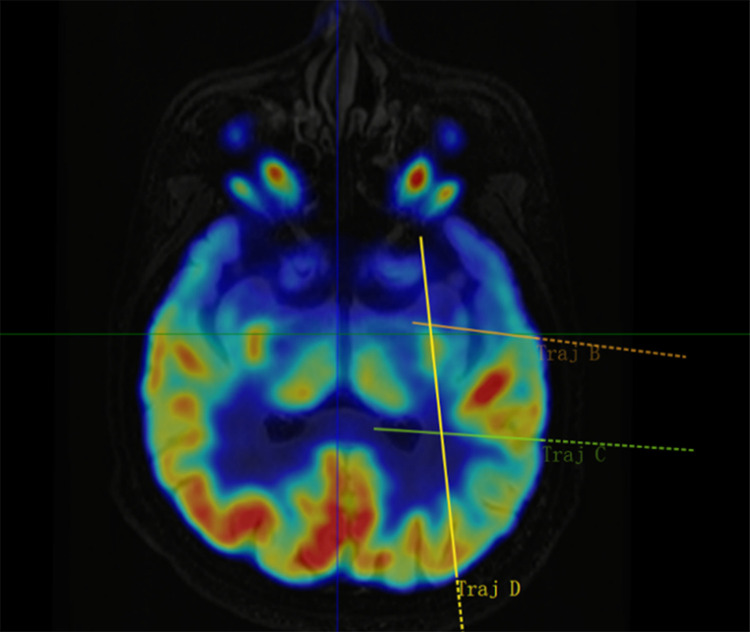
Co-registered axial MRI T2 FLAIR and PET-CT images.

**Fig 4:**
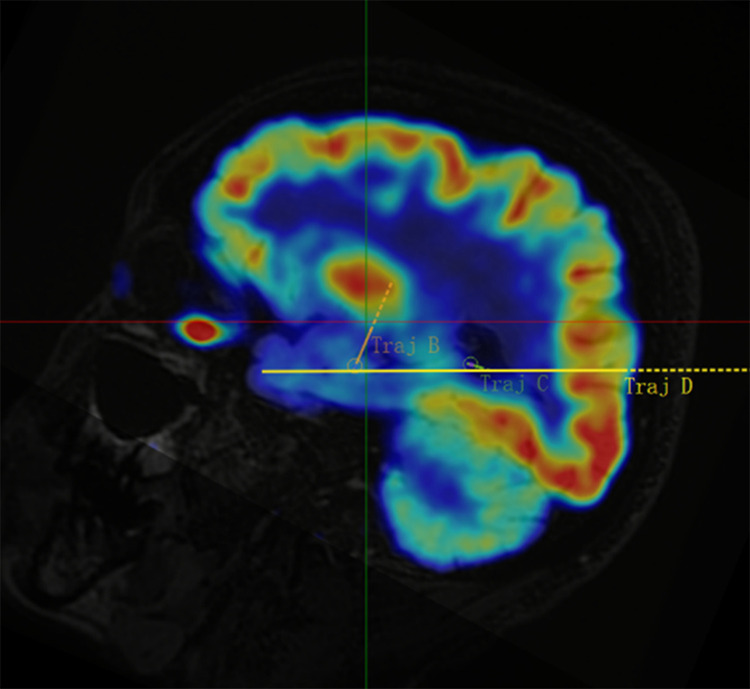
Co-registered sagittal MRI T2 LAIR and PET-CT images.

**Fig 5:**
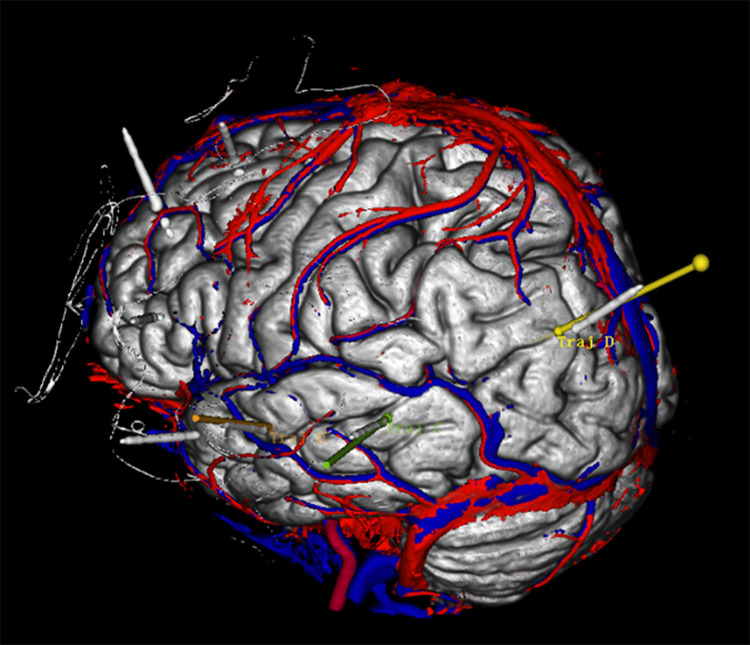
Three-dimensional reconstruction based on CT and MRI images after SEEG electrode implantation. Legend: The target at the tip of electrode B is the hippocampus head, the target at the tip of electrode C is the hippocampus tail, and the target at the tip of electrode D is the amygdala. Additionally, the target in the middle segment of electrode D is the hippocampus body. Fig 5 Cerebral arteries are marked in red, cerebral veins in blue, and the implanted electrodes (B, C, D) are represented by colored cylinders (other SEEG electrodes are shown in gray).

Figs 1–5 display the pre- and post-implantation imaging of a patient with hippocampal-origin epilepsy and comorbid depression who underwent SEEG electrode placement.

### Statistical analysis

Statistical analyses were performed using SPSS 26.0 software. For continuous variables, group comparisons were performed using an independent sample t-test (or Levene’s test if variances were unequal). Categorical data were expressed as counts and percentages; group comparisons were made using the chi-square (χ²) test (or Fisher’s exact test if any expected cell frequency was < 5). Variables with a P-value < 0.05 in the univariate analysis were considered statistically significant and were further entered into a linear Lasso regression model to identify independent risk factors. R software (download available at http://www.r-project.org) was then used to construct a nomogram model based on the identified independent risk factors. The model’s accuracy was estimated using the concordance index (C-index), and ROC curves along with the area under the AUC were plotted to evaluate the model’s discriminative ability and predictive accuracy. Calibration curves were plotted using the “Boot” method to assess the model’s performance.

## Results

### Univariate analysis of risk factors for comorbid depression in epilepsy

A total of 170 epilepsy patients were initially identified from medical records. After applying exclusion criteria, 11 cases were excluded due to unavailable C-NDDI-E results to impaired consciousness or cognitive dysfunction, 7 cases were excluded for comorbid malignancies. Finally, 152 patients were included, with 43 in the study group and 109 in the control group. The mean age of the study group was 32.40 ± 15.06 years, compared to 43.28 ± 14.32 years in the control group, and this difference was statistically significant (p < 0.001). The proportion of males was 53.5% in the study group and 58.7% in the control group, showing no significant difference (p = 0.557). Significant intergroup differences were found for employment status (p = 0.009), marital status (p < 0.001), age at onset (p = 0.030, 95% CI: –1.602 to –0.082), frequency of epileptic seizures (p = 0.002), types of drug treatment (p = 0.004), scalp EEG-determined epileptogenic zone (p = 0.014), SEEG-determined epileptogenic zone (p = 0.048), and ADL score (p = 0.001, 95% CI: –8.686 to –2.344). The remaining factors showed no statistically significant differences between the groups (p > 0.05). See Table 1

**Table 1:** Univariate analysis results for comorbid depression in epilepsy.

Factors	Study group	Control group	t/χ²	p	95% confidence interval
Number	43	109			
Average age	32.400 ± 15.056	43.280 ± 14.324	−4.157	<0.001	(−16.051, −5.709)
Male proportion	23/53.5%	64/58.7%	0.344	0.557	
BMI	21.998 ± 2.127	21.258 ± 3.903	1.175	0.242	(−0.505, 1.984)
Education level (primary and below)	15/34.9%	23/21.1%	7.630	0.178	
Employment status (with a steady income)	9/21.0%	38/30.0%	15.387	0.009	
Marital status (divorce, widowed)	9/20.9%	1/0.9%	26.837	<0.001	
Age of onset	7.791 ± 1.934	8.633 ± 2.210	−2.190	0.030	(−1.602, −0.082)
Frequency of epileptic seizures per month (>4 times)	17/39.5%	18/16.5%	9.220	0.002	
Drug treatment (1^a^)	17/39.5%	19/17.4%	8.335	0.004	
topiramate	5/11.6%	4/3.7%			
phenobarbital	12/28.0%	15/13.8%			
levetiracetam	1/2.3%	2/1.8%			
perampanel	0/0	1/0.9%			
Scalp EEG-determined epileptogenic zone			10.669	0.014	
Temporal lobe	26/60.5%	38/34.9%			
Frontal lobe	6/14.0%	12/11.0%			
Parietal lobe	8/18.6%	38/34.9%			
Occipital lobe	3/7%	21/19.3%			
Number of epileptogenic zone (multiple zone)	15/34.9%	22/20.2%	3.618	0.057	
SEEG-determined epileptogenic zone			4.250	0.039	
Hippocampal	11/64.7%	5/29.4%			
Non-hippocampal	6/35.3%	18/31.3%			
Family history of epilepsy or depression	2/4.7%	7/6.4%	0.174	0.677	
ADL score	82.558 ± 10.022	88.073 ± 8.441	−3.436	0.001	(−8.686, −2.344)

^a^The medication regimen includes topiramate, phenobarbital, levetiracetam or perampanel.

### Multivariate analysis of risk factors for comorbid depression in epilepsy

Factors with p-values < 0.05 in the univariate analysis—namely age, employment status, marital status, age at onset, frequency of epileptic seizures, types of drug treatment, SEEG-determined epileptogenic zone, scalp EEG-determined epileptogenic zone, and ADL score—were entered into a binary logistic regression model. In addition, the number of epileptogenic zone (p = 0.057), although not significant at the conventional 0.05 level, demonstrated borderline significance (p < 0.1) and was therefore included for further evaluation.

The multivariate binary Lasso regression analysis indicated that marital status (p = 0.0008), Enrollment age (OR = 0.9152, P = 0.0003, 95% CI: 0.8673–0.9562), frequency of epileptic seizures (OR =5.9946, P = 0.0030, 95% CI: 1.8952–20.6541), type of drug treatment (OR = 44.4062, P = 0.0157, 95% CI: 1.3629–15.6702), SEEG-defined epileptogenic zone (hippocampal onset: OR = 12.3489, P = 0.0026, 95% CI: 2.5902–70.9811), and ADL score (OR = 0.9358, P = 0.0314, 95% CI: 0.8785–0.9930) were independent risk factors for comorbid depression in epilepsy. However, the scalp EEG-determined epileptogenic zone (p = 0.0844) and employment status (p = 0.1750) were not independent risk factors.

Although age of onset (p = 0.0626) and the number of epileptogenic zone (p = 0.3142) did not reach statistical significance, they exhibited a trend toward being risk factors for comorbid depression in epilepsy. See Table 2

**Table 2:** Results of linear Lasso regression analysis for comorbid depression in epilepsy.

Factors	p	OR value	95% confidence interval	
			lower limit	upper limit
Enrollment age	0.0003	0.9152	0.8673	0.9562
Employment status	0.1750	0.3881	0.0892	1.4141
Marital status (stable)	0.0008	50.5132	7.0107	1060.4966
Age of onset	0.0626	0.7806	0.5900	0.9995
Frequency of epileptic seizures per month	0.0030	5.9946	1.8952	20.6541
Drug treatment	0.0157	4.4062	1.3629	15.6702
Scalp EEG-determined epileptogenic zone (Temporal lobe)	0.0844	2.5937	0.8929	7.9732
SEEG-determined epileptogenic zone (Hippocampal)	0.0026	12.3489	2.5902	70.9811
ADL score	0.0314	0.9358	0.8785	0.9930
Number of epileptogenic zone	0.3142	1.8256	0.5599	5.9904

### Development of a nomogram model for predicting the risk of comorbid depression in epilepsy

**Fig 6:**
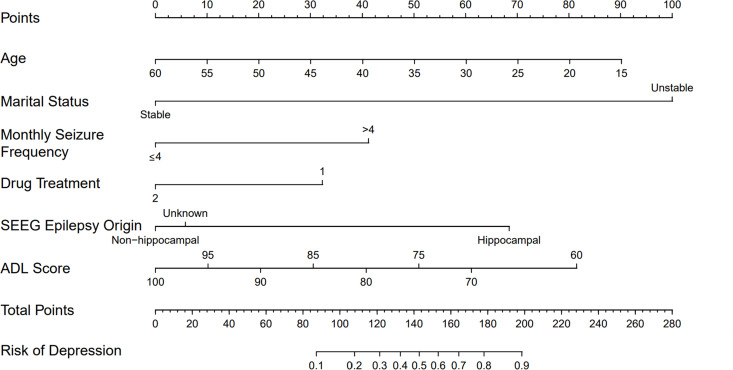
Prediction model for the risk of comorbid depression in epilepsy. Note: Type of Medication. 1. The medication regimen includes topiramate, phenobarbital, levetiracetam or perampanel. 2. The medication regimen does not include the aforementioned drugs.

Using R software, a Lasso regression model was established, and a nomogram was constructed. Among the results of the multivariate Lasso regression analysis, 6 factors were found to be statistically significant. Based on these six factors, a nomogram model was developed to predict the risk of comorbid depression in epilepsy, as shown in Fig 6. In Fig 6, locate the corresponding point on the axis for each variable. Draw a vertical line from this point to intersect with the scoring scale above, which gives the score for that variable. Sum the scores of all variables to obtain the total score, and use this total score to determine the risk of comorbid depression in epilepsy.

### Accuracy assessment of the nomogram model for predicting the risk of comorbid depression in epilepsy

**Fig 7:**
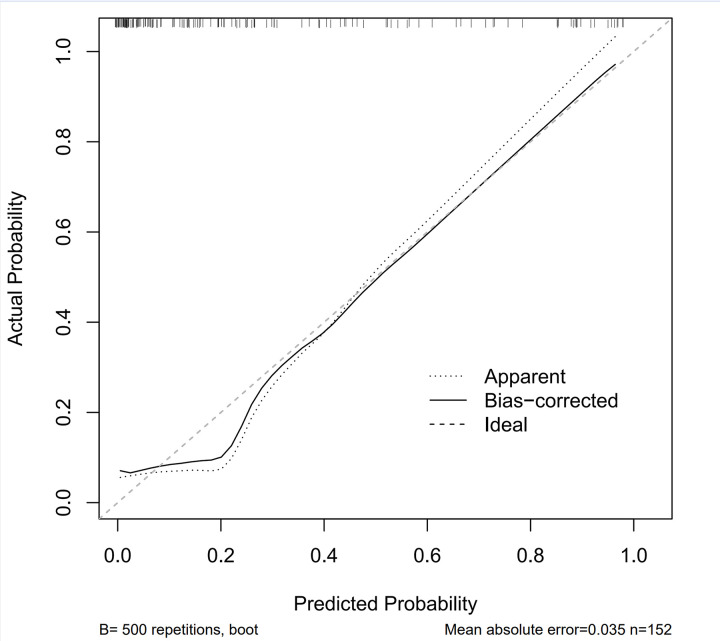
Calibration curve of the Lasso regression model.

The calibration curve was plotted using the “Boot” method. The calibration curve is used to evaluate the accuracy of the model’s predicted probabilities. The X-axis represents the predicted probabilities, while the Y-axis represents the observed probabilities. The relationship between the model’s predicted probabilities and the actual observed probabilities is close to the ideal scenario. The bias-corrected curve is even closer to the ideal curve, indicating good calibration of the model and high accuracy in predicting risk. As in Fig 7.

### ROC curve of the nomogram model for predicting the risk of comorbid depression in epilepsy

**Fig 8:**
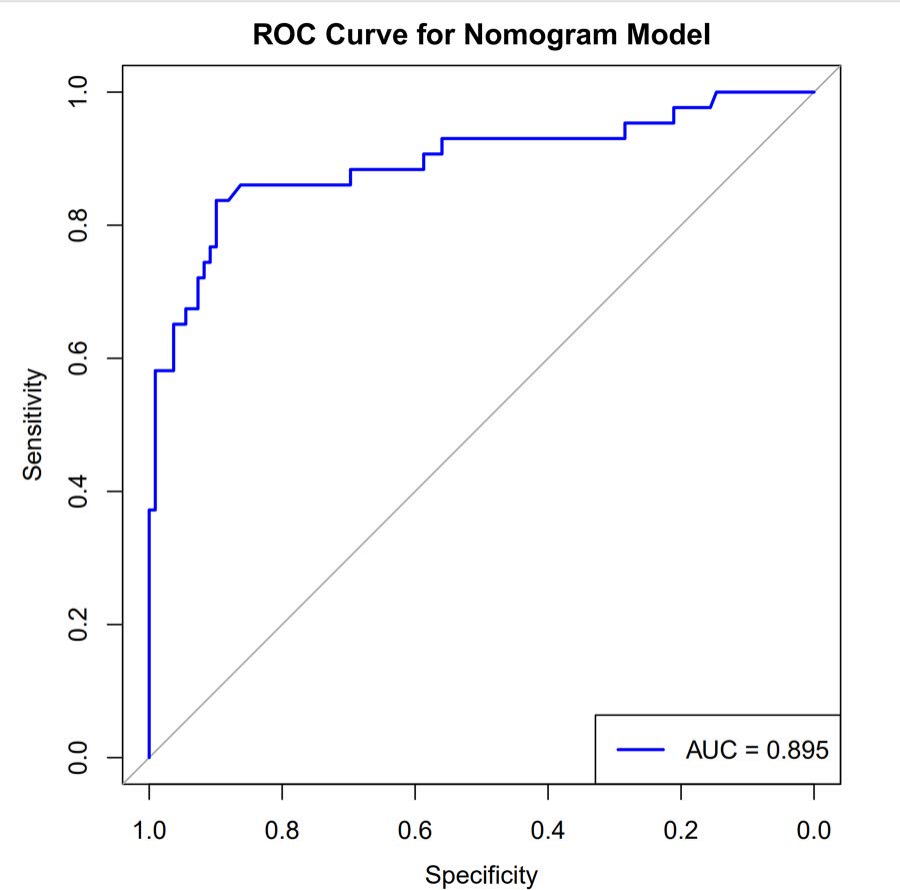
ROC curve of the nomogram model for predicting the risk of comorbid depression in epilepsy C-index: 0.895.

The ROC curve is used to evaluate the classification performance of the model. The closer the AUC value is to 1, the stronger the model’s discriminative ability. An AUC value of 0.895 indicates that the model has a high discriminative ability. The C-index is an indicator that measures the consistency between the model’s predicted results and the actual outcomes. The closer the C-index value is to 1, the higher the predictive accuracy of the model. A C-index value of 0.895, which is close to 1, indicates that the model has high accuracy in predicting ordered categorical outcomes. This ROC curve demonstrates that the model performs well in classification tasks, with high discriminative ability and predictive accuracy. As in Fig 8.

## Discussion

In recent years, the comorbidity of epilepsy and depression has increasingly attracted the attention of clinicians and researchers. The incidence of depression in patients with epilepsy is significantly higher than that in the general population [16]. Among epilepsy patients, the prevalence of depression is approximately 20%–50% [25–28], whereas in the general population it usually ranges between 5% and 10% [29,30] Studies have shown that the comorbidity of epilepsy and depression not only affects patients’ quality of life but may also negatively impact the treatment and management of epilepsy, thereby exacerbating patients’ symptoms and prognosis [31]. Numerous studies have identified various risk factors for comorbid depression in epilepsy, including the type of epilepsy, seizure frequency, duration of seizures, adverse effects of drug treatments, and the patient’s social support system [32]. In particular, certain types of epilepsy, such as temporal lobe epilepsy and post-traumatic epilepsy, are associated with a higher risk of comorbid depression [33]. Furthermore, neurobiological factors such as neurotransmitter imbalances [34,35], decreased levels of growth hormone [36], structural brain changes [37], and inflammatory responses [38] have also been closely linked to the comorbidity of epilepsy and depression [30] Given the current lack of awareness and the low diagnostic rate of this comorbidity in clinical practice [25], we combined SEEG data from our hospital to develop a predictive model for the risk of developing comorbid depression in epilepsy, aiming to improve the identification and management of this patient population.

In this study, we analyzed clinical data from 152 epilepsy patients and, in conjunction with the epileptogenic zone obtained from SEEG and 13 other factors, identified the independent risk factors for comorbid depression in epilepsy. We subsequently developed a nomogram model to predict the risk of depression in epilepsy patients. The results indicated that marital status (with divorce or widowhood significantly increasing the risk, p = 0.0008), enrollment age (with younger age enrollment associated with a higher risk, OR = 0.9152, p = 0.0003), seizure frequency (more than 4 times per month, with a markedly increased risk, OR = 5.9946, p = 0.0030), types of drug treatment (regimens including topiramate, phenobarbital, levetiracetam, or perampanel significantly increased the risk, OR = 4.4062, p = 0.0157), SEEG-determined epileptogenic zone (especially in patients with hippocampal onset, who demonstrated a markedly increased risk, OR = 12.3489, p = 0.0026), and ADL score (lower scores indicating a higher risk, OR = 0.9358, p = 0.0314) were independent risk factors for comorbid depression in epilepsy.

The nomogram model established in this study exhibited high discriminative ability and predictive accuracy (AUC = 0.895, C-index = 0.895). In SEEG results, the hippocampus being the origin of epilepsy, being divorced or widowed, younger age of enrollment, higher frequency of epileptic seizures (>4 times/month), the type of drug treatment (including regimens with topiramate, phenobarbital, levetiracetam, and perampanel) and lower ADL scores were associated with an increased risk of depression. While our findings align with established risk factors (younger age of onset, social isolation, and high seizure frequency) reported in prior studies, this study advances the field by quantifying their combined predictive power through a clinically implementable nomogram. The nomogram’s clinical utility lies in its ability to stratify depression risk using routine clinical data, complementing standardized screening tools like the NDDI-E. Clinicians can adopt a stepped-care approach: Initial risk stratification: Apply the nomogram to identify high-risk patients (e.g., those with younger age of onset, hippocampal-onset seizures, frequent seizures, or low ADL scores); Targeted screening: Prioritize these patients for NDDI-E administration to confirm depressive symptoms; Integrated intervention: For screen-positive cases, combine seizure control optimization (e.g., adjusting topiramate/levetiracetam regimens) with psychiatric referrals and social support programs for divorced/widowed patients. This dual strategy may improve detection rates compared to universal NDDI-E screening alone, particularly in resource-limited settings. For identified high-risk patients, we propose: Pharmacological: Avoid depression-aggravating drug treatment (e.g., topiramate, phenobarbital, levetiracetam, and perampanel) in favor of mood-neutral alternatives (e.g., lamotrigine [39]), where clinically appropriate; Psychosocial: Integrate routine mental health assessments during epilepsy clinic visits for patients with ≥2 risk factors; System-level: Develop automated alerts triggered by nomogram risk scores (e.g., > 70%) to prompt clinician evaluation.

Despite these promising results, several limitations of this study should be acknowledged. 1. the sample size was relatively small (152 cases), and all patients were recruited from a single medical institution, which may introduce selection bias and limit the generalizability of the findings. 2. as a cross-sectional study, it did not explore the causal relationship between epilepsy and comorbid depression; future longitudinal studies are needed to further verify the long-term impact of these risk factors. Additionally, certain potentially important variables, such as patients’ psychosocial support and medication adherence, were not included in the model, although these factors may significantly influence the risk of depression. 3. this study was primarily based on SEEG and EEG findings without incorporating other neuroimaging or biomarker data; future research should consider integrating multimodal data to enhance the predictive capability of the model. 4.This study did not match participants for demographic or clinical features known to influence depression risk in the general population (e.g., age, gender, family history of depression, trauma history), which may introduce confounding effects. Future prospective, matched studies are warranted to more precisely identify epilepsy-specific risk factors for comorbid depression. 5. The diagnosis of depression in our study relied on the C-NDDI-E scale and a clinical symptom checklist, without the use of structured psychiatric interviews (e.g., MINI, SCID) or formal DSM-5-based assessments. Given the retrospective nature of the study, such tools were not feasible. Therefore, the possibility of diagnostic misclassification cannot be excluded. Future prospective studies incorporating standardized psychiatric evaluations are warranted to enhance diagnostic precision. 6. The aggregation of AEDs with potential depressogenic effects (topiramate, levetiracetam, phenobarbital, perampanel) was necessary due to sample size constraints. While this precludes drug-specific inferences, it reflects real-world polypharmacy patterns and provides clinically actionable evidence on high-risk regimen profiles. Future large-scale pharmacovigilance studies are warranted to dissect individual AED contributions. 7. Internal validation, though essential, isn’t enough to prove how well our model works in different situations. We really need external and temporal validation to check its performance across diverse settings and over time. 8.Our model did not capture psychosocial dimensions (e.g., perceived stigma, social isolation, economic stress, family support) known to influence depression in epilepsy. While this reflects real-world clinical data constraints, it risks omitted variable bias and limits mechanistic interpretation. Future studies should integrate standardized instruments like the Stigma Scale for Epilepsy to quantify these complex interactions.

Future research should aim to expand sample sizes and incorporate multicenter prospective cohort studies to enhance the representativeness and generalizability of findings. Additionally, integrating multimodal data such as neuroimaging and genetic testing may deepen our understanding of the biological mechanisms underlying the comorbidity of epilepsy and depression, providing more precise diagnostic strategies. In this study, NDDI-E scores were used to dichotomize patients into depressed and non-depressed groups based on a validated cutoff (>12). Future studies could further explore the relationship between risk factors and the severity of depressive symptoms using continuous NDDI-E scores. Furthermore, developing multidisciplinary interventions that combine pharmacological treatment, psychological support, and social care is critical to improving patient outcomes.

## Supporting information

S1 File Ethics committee approval letter of biomedical research involving humans. (DOCX)

S2 File Ethics committee approval letter of biomedical research involving humans-chinese version. (PDF)

S3 File Human participants research checklist. (DOCX)
