# Supplementary material for: Risk factor analysis and establishment of a predictive model for epilepsy comorbid with depression
Source: PLoS One. 2025 Sep 2;20(9):e0331441. doi: 10.1371/journal.pone.0331441 (PMC12404384; doi:10.1371/journal.pone.0331441)
Supplement: S1 File — (DOCX) [file pone.0331441.s001.docx]

**Ethics Committee Approval Letter of Biomedical Research Involving Humans**

**Approval No. : Zhejiang Tongde Lunshen 2025 Yan No. 094 - JY**

| **Study Title** | | **Analysis of Risk Factors and Establishment of a Prediction Model for Epilepsy-Depression Comorbidity** | |
| --- | --- | --- | --- |
| **Sponsor** | | **Tongde Hospital of Zhejiang Province** | |
| **Acceptance Number** | | **2025 - 074 (K)** | |
| **Principal Investigator** | **Sun Yiming** | **Department** | **Department of Neurosurgery** |
| **Category of Review** | **Initial Review** | **Type of Review** | **Simplified Review** |
| **Date of Review** | **February 27, 2025** | **Location of Review** | **Room No. 1, Building No. 6** |
| **Items Reviewed :** | | **1. Initial Review Application**  **2. Principal Investigator's Resume and Investigator's Commitment Letter**  **3. Clinical Trial Protocol (Version No.: V1.0 Version Date: December 13, 2024)**  **4. Application for Waiver of Informed Consent**  **5. Data Collection Form (Version No.: V1.0 Version Date: February 24, 2025)** | |
| **Evaluation** | | **The Ethics Committee has conducted an initial review of the above-mentioned documents and considers that the clinical trial protocol is basically in compliance with ethical standards. The committee agrees to implement the clinical research.** | |
| **Decision** | | **The committee's review decision is: Approval** | |
| **Chair Signature** | |  | |
| **Date of Issue** | | **February 2025** | |
| **Stamp of Ethics Committee** | | **[Ethics Committee Stamp]** | |
| **Period of Validity** | | **From the date of initial review approval by this Ethics Committee, the clinical research should be initiated in this hospital. If not initiated within the time limit, this approval will automatically become void.** | |
| **Continue Review** | | **The review frequency is once every 12 months from the date of approval of this study. For the first time, please submit a research progress report one month before February 26, 2026. The Ethics Committee reserves the right to change the frequency of follow-up reviews based on the actual progress of the study.** | |
| **Statement** | | **The responsibilities, composition, operating procedures, and records of this Ethics Committee follow the "Ethical Review Measures for Biomedical Research Involving Humans," the "International Ethical Guidelines for Health-Related Research Involving Humans," the "Declaration of Helsinki," GCP, and ICH-GCP, as well as relevant domestic laws and regulations.** | |
| **Notes :**  **1. Please follow the ethical principles in relevant Chinese laws, regulations, and rules.**  **2. Please follow the clinical research protocol, informed consent form, and recruitment materials approved by this Ethics Committee to conduct this study and protect the health and rights of the subjects. Any modifications to the research protocol, informed consent form, and recruitment materials must be reviewed and approved by this Ethics Committee before implementation.**  **3. SAE/SUSARs occurring in this hospital, as well as safety updates during the research and development period, must be submitted to this Ethics Committee in a timely manner according to the latest requirements of NMPA/GCP. SAE/SUSARs occurring in other centers domestically and internationally should be regularly summarized and assessed before being submitted to this Ethics Committee.**  **4. Based on the report, this Ethics Committee has the right to make new decisions upon assessment.**  **5. Starting from today, regardless of whether the study has begun or not, please submit a research progress report one month before the follow-up review date expires.**  **6. The sponsor should submit a summary of the research progress report to the lead unit's ethics review committee; if any situation that may significantly affect the progress of the study or increase the risk to the subjects occurs, the applicant should promptly submit a written report to this Ethics Committee.**  **7. If the study includes subjects who do not meet the inclusion criteria or meet the exclusion criteria, fails to withdraw subjects in accordance with the termination regulations, provides incorrect treatment or dosage, or administers prohibited concomitant medications as specified in the protocol, or other situations that do not follow the protocol and may adversely affect the rights or health of the subjects or the scientific nature of the study, the sponsor, monitor, or investigator should submit a protocol deviation report.**  **8. If the applicant suspends or prematurely terminates the clinical research, please promptly submit a suspension or termination report.**  **9. Upon completion of the clinical research, please submit a final report.**  **10. Any research project involving the collection of samples or data related to Chinese human genetic resources must obtain approval from the Office of the Administration of Human Genetic Resources of China before conducting the study in this center.**  **11. Any research project approved by this Ethics Committee must be registered on the clinical research registration and filing information system platform of the National Health Commission, the Center for Drug Evaluation, etc., in accordance with relevant regulations before implementation.** | | | |
